# Hydroxycarbamide and Sickle Cell Anemia: Paradoxical Effects Related to Redox Mechanisms of Cellular Adaptation

**DOI:** 10.1021/acsomega.5c07897

**Published:** 2025-12-09

**Authors:** Ilana Luize Rocha Santana, Victoria Simões Bernardo, Edis Belini Junior, Pâmela Lourdes Pereira da Silva, Danilo Grünig Humberto da Silva, Larissa Paola Rodrigues Venancio

**Affiliations:** † Department of Biology, 28108 Universidade Estadual Paulista Julio de Mesquita Filho, São José do Rio Preto, Sao Paulo 01049-010,Brazil; ‡ 54534 Universidade Federal de Mato Grosso do Sul, CPTL/UFMS Três Lagoas, Mato Grosso do Sul 79600-080,Brazil; § 423876 Universidade Federal do Oeste da Bahia, Center for Biological and Health Science Barreiras, Bahia 47808-021,Brazil

## Abstract

Sickle cell anemia (SCA) is a hemolytic anemia characterized by a chronic redox imbalance with few disease-modifying treatments available. In this study, we aimed to determine the influence of hydroxycarbamide (HC) treatment on the redox mechanisms of cellular adaptation in patients with SCA. We analyzed 10 patients treated (HC+) and 9 not treated (HC−). We evaluated, by RT-qPCR, the transcript levels of the transcription factors *NRF2* and *ATF4*, their modulators (*KEAP1*, *AKT*, and *PI3K*), and the antioxidants (*SOD1*, *CAT*, *PRDX1*, and *GPX1*). We also collected data on biochemical parameters, including total leukocyte count, direct and indirect bilirubin, lactic acid dehydrogenase (LDH), and fetal hemoglobin (HbF%) levels, by analyzing the participants’ medical records. Among the results obtained, *NRF2* and *AKT* presented increased mRNA levels in the HC+ group, but reduced *ATF4*, *CAT*, and *SOD1* mRNA levels. Multivariate statistical analysis ranked *NRF2* and *ATF4* as the markers that most characterized the groups studied. Therefore, taking together our results and the literature, we suggest that HC use results in a dynamic relationship between the redox signaling pathways of the investigated transcription factors triggered as part of an adaptive response to this medication, associated with their involvement in Hb F production.

## Graphical abstract

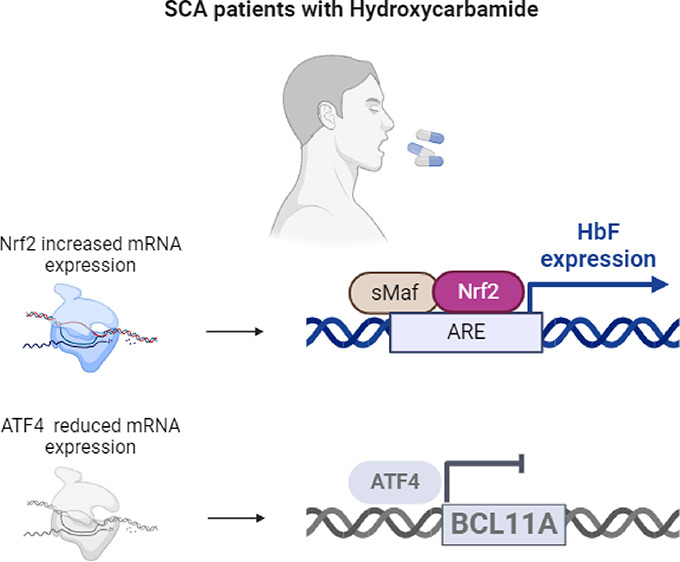

## 1 Introduction

Sickle cell anemia (SCA) is a hemolytic anemia characterized by a point mutation (*HBB*:c.20A > T; rs334) in homozygosis that produces the hemoglobin S (HbS).^1–4^ The HbS is responsible for most of the reactive oxygen species (ROS) generated in these cells (approximately double the amount compared to the normal red blood cells), causing extensive oxidative damage.^5,6^ Despite its well-defined molecular characterization, SCA presents many complications, such as chronic inflammation and oxidative stress,^7^ resulting in a clinical heterogeneity that is not fully understood.^8–11^ Thus, redox homeostasis disruption forms an essential part of the complex pathology of this disease, with signs of oxidative stress observed in various tissues.^7^ Therefore, highlighting the importance of redox biology in SCA and emphasizing that studies that aim to understand this disease’s redox signaling network are crucial.^12–14^ In this scenario, nuclear factor erythroid 2-related factor 2 (NRF2) is one of the central players controlling the oxidative stress response.^15^

Under oxidative stress, NRF2 translocates to the nucleus. It initiates the transcription of numerous antioxidant genes with the antioxidant response element (ARE) promoter, such as superoxide dismutase 1 (*SOD1*), catalase (*CAT*), glutathione peroxidase 1 (*GPX1*), and peroxiredoxin 1 (*PRDX1*).^16,17^ Under physiological conditions, NRF2 is degraded through the ubiquitin-proteasome pathway, a mechanism triggered by the Kelch-like ECH-associated protein1 (KEAP1), an oxidative stress sensor.^18^ Unlike what we observed for KEAP1, other elements, such as protein kinase B (PKB/AKT) and phosphoinositide 3-kinase (PI3K), stabilize NRF2 and, consequently, suppress NRF2’s degradation.^15,18^ Therefore, as a master regulator of the antioxidant processes, the NRF2/ARE signaling pathway is protective in multiple diseases with redox imbalance conditions, such as SCA.^19^

Unfortunately, few disease-modifying agents that target the underlying pathophysiology of SCA exist. Moreover, even fewer are approved for use. The first Food and Drug Administration (FDA)-approved drug (and still the primary medication used to date) was hydroxycarbamide (HC), a compound that decreases intracellular HbS polymerization.^20^ This oral drug has been shown to have many other beneficial effects for treating SCA, including improvement of sickle cell metabolism and reducing inflammation and oxidative stress.^21,22^ However, despite decades of study, HC’s action mechanism still needs to be fully understood to explain the different patients’ responses to its use, which fascinates scientists today.^23^

Recently, Santana et al. provided evidence that HC directly neutralizes free radicals and stimulates the production of antioxidant genes via activating the NRF2 signaling pathway, using human peripheral blood mononuclear cells (PBMCs) and human umbilical vein endothelial cells (HUVECs).^24^ In addition, Huang et al. identified transcription factor activating transcription factor 4 (ATF4), a known heme-regulated inhibitor (HRI)-regulated protein, as a novel gamma (γ)-globin regulator. ATF4 directly stimulates the transcription of *BCL11A*, a repressor of γ-globin transcription, by binding to its enhancer and fostering enhancer-promoter contacts.^25^ Furthermore, mRNA level analysis of patients with SCA during vaso-occlusive crises and in a steady state, compared with age-matched healthy patients, highlights both *NRF2* and *ATF4* differential expressions, indicating that they are possible candidates involved in the severity of this pathology.^26^

Thus, observing the beneficial effects of HC in the treatment of individuals with SCA and viewing the scarce knowledge involving its influence on redox homeostasis, this study aimed to determine the influence of HC treatment on critical pathways of redox adaptation in SCA patients and their association with biochemical disease modifiers and transcript levels on reticulocytes.

## 2 Results

### 2.1 Transcript Levels of the Redox Signaling Pathways

We found statistically significant reduced mRNA levels of *ATF4* (Figure 1A), *SOD1* (Figure 2A), and *CAT* (Figure 2B) transcripts of 2-, ∼1.3-, and ∼1.5-fold, respectively, in the HC^+^ group compared with HC^–^. Additionally, mRNA levels of *NRF2* (Figure 1B) and *AKT (* Figure 1C) increased, respectively, by ∼3- and ∼9-fold in the HC^+^ group compared to HC^–^. More details about these analyses are in Table S2, Supporting Information.

**Figure 1:**
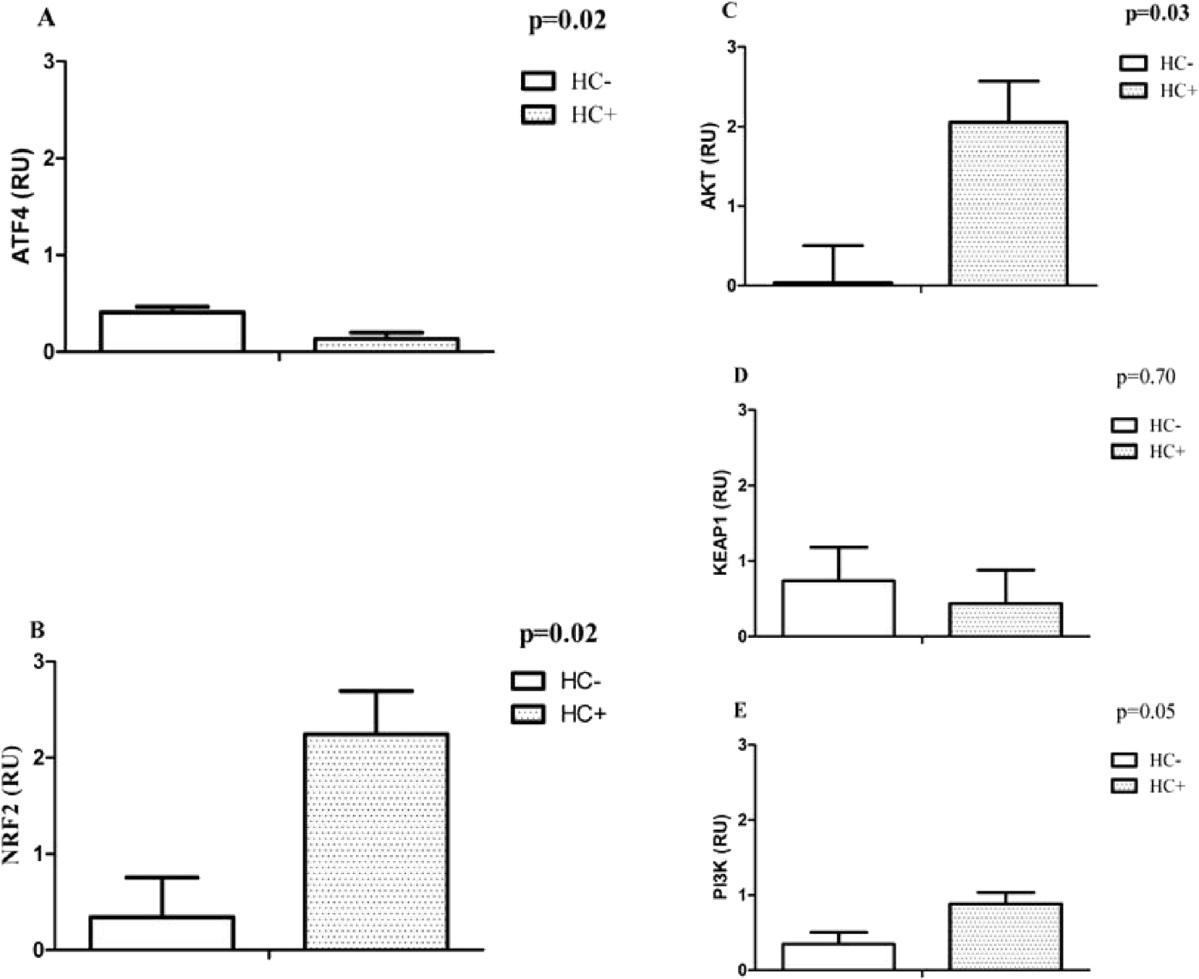
Relative mRNA levels of the transcription factors and their modulators in reticulocytes. Results were generated using ANCOVA analysis (correcting for age, sex, and HC dose). Data expressed as mean ± standard error of mean (SEM). *P* < 0.05 was considered to be statistically significant (in bold). (A) Activating transcription factor 4 (ATF4). (B) NF-E2 p45-related factor 2 (NRF2). (C) Protein kinase B (PKB/AKT). (D) Kelch-like ECH-associated protein 1 (KEAP1). (E). Phosphoinositide-3-kinase (PI3K). RU - relative expression.

**Figure 2:**
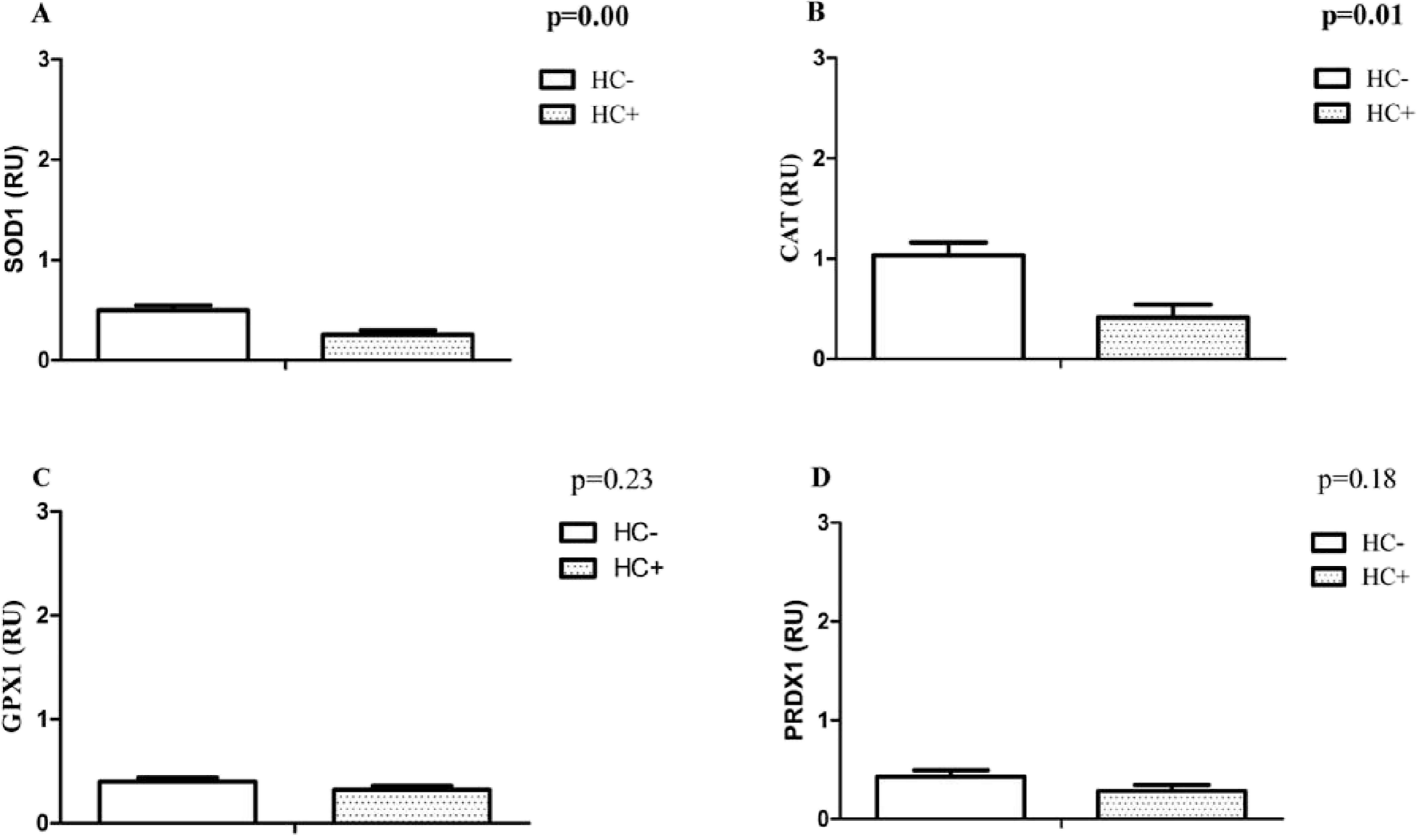
Relative mRNA levels of essential antioxidants involved in redox adaptation in reticulocytes. Results were generated using ANCOVA analysis (correcting for age, sex, and HC dose). Data expressed as mean ± standard error of mean (SEM). *P* < 0.05 was considered to be statistically significant (in bold). (A). Superoxide dismutase 1 (SOD1). (B) Catalase (CAT). (C) Glutathione peroxidase 1 (GPX1). (D) Peroxiredoxin 1 (PRDX1).

Therefore, it is possible to assume that the NRF2 pathway is active in the HC^+^ group. However, the absence of an increase in the transcripts of the antioxidant enzymes evaluated suggests that its central role did not involve ROS resistance in reticulocytes (Figure 2)—more details about these analyses are in the Supporting Information.

### 2.2 Metabolic Separation of Sickle Cells with or without HC Use

The PLS-DA was employed as a discrimination tool to investigate whether the HC+ group can be effectively distinguished from the HC^–^ group by investigating the data distribution of all of the evaluated biomarkers.

The results of the multivariate analysis were consistent with the study’s strict inclusion/exclusion criteria, as well as the wide variation in responses to HC use reported in the literature. In other words, most HC^+^ individuals (“green dots”) clustered apart from the HC^–^ group (“red dots”). However, some overlap was observed, in agreement with the variability in response to HC described in the literature (Figure 3A). Components 1 and 2 explained 11.8 and 15.9% of the variance, respectively, which, although modest, were sufficient to reveal group separation. Moreover, the analysis identified the most critical features ranked (>1) based on the projection of important variable (VIP) scores that contributed to validating the biomarkers evaluated (Figure 3B) and to the separation of the groups mentioned above, reinforcing the univariate observations described previously. Therefore, the transcription factor levels were the variable that most accurately depicted the studied groups, with statistically significant differences observed in the univariate analysis. In Figure 3C, the most influential data points are located in the outermost areas along the direction of separation, as identified in the corresponding score plot.

**Figure 3:**
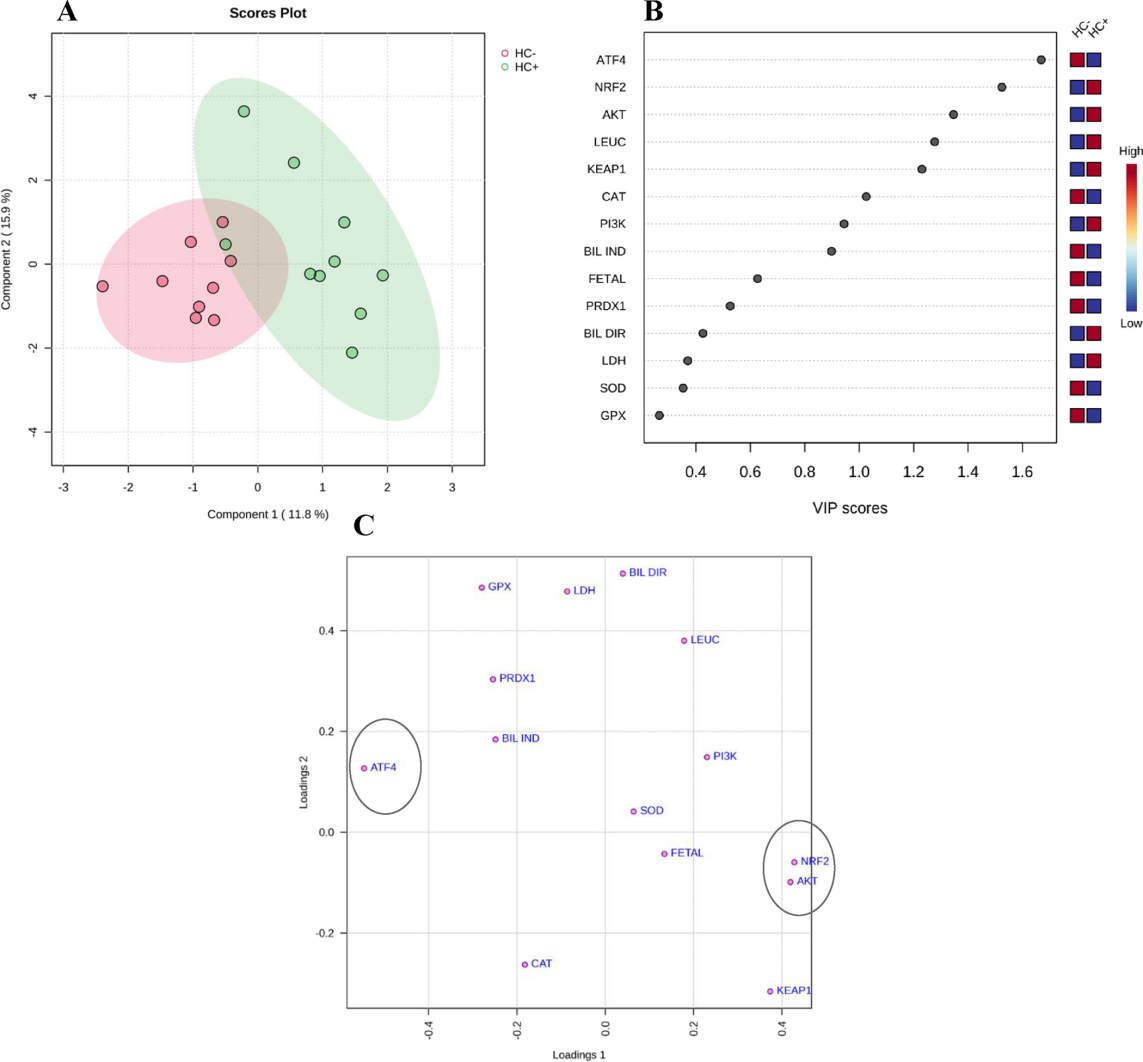
PLS-DA score plot of the two principal components. (A) Shaded elliptical areas indicate regions with 95% confidence. Green dots indicate patients using HC (HC^+^), while red dots indicate patients not using the drug (HC^–^) (B). VIP score ranked the most important features. On the *y*-axis, in the left corner of the image, all of the variables studied for the groups (HC^+^ vs HC^–^) are shown. The analyzed genes are activating transcription factor 4 (ATF4), protein kinase B (PKB/AKT), phosphoinositide-3-kinase (PI3K), NF-E2 p45-related factor 2 (NRF2), Kelch-like ECH-associated protein 1 (KEAP1), peroxiredoxin 1 (PRDX1), superoxide dismutase 1 (SOD1), glutathione peroxidase 1 (GPX), and catalase (CAT), and the biochemical parameters are fetal hemoglobin (FETAL), leucocytes (LEUC), direct bilirubin (DIR BIL), lactate dehydrogenase (LDH), and indirect bilirubin (IND BIL). The mini heatmap on the right represents concentration variations within groups. (C) The loadings plot of the first two principal components shows all variables of the study. The circles indicate the most influential data points in the corresponding score plot. Source: Metaboanalyst.

### 2.3 Association Degree Results

We investigated the relationship between the mRNA levels of transcription factors and their modulators, the antioxidants, and the HbF levels to expand our understanding of the redox homeostasis contribution to the SCA phenotypic variability. We verified whether the data distribution of the transcript factors and their modulators (as independent variables) had any effect on the expression of their downstream target genes and HbF levels. We did not find any association (Table 1).

**Table 1:** Regression Model Analysis^a^

dependent variable	independent variable
	ATF4/NRF2/AK*T*/KEAP1/PI3K
Hb F%	*R* = 0.85; *p* = 0.35
CAT	*R* = 0.74; *p* = 0.24
SOD1	*R* = 0.72; *p* = 0.22
GPX1	*R* = 0.83; *p* = 0.05
PRDX1	*R* = 0.49; *p* = 0.74

a We analyzed the association degrees of mRNA levels of transcription factors and their modulators with the antioxidants and the HbF levels. The dependent variables were antioxidants and HbF levels, while the independent variables included transcription factors and their modulators. *R*: multiple association coefficient (general regression model analysis, multiple regression design); *p* < 0.05 was considered statistically significant.

## 3 Discussion

HC was the first and only FDA-approved drug for treating SCD from 1998 to 2017.^30^ Notably, while other drugs have been studied over the past decades, HC is still considered the pillar for the pharmacological treatment of SCD due to its multifactorial protection mechanism for sickle RBC alterations. HC is a compound with a multifactorial mechanism of action whose beneficial effects are associated with several underlying pathophysiology of this disease, including an increase in the cell’s antioxidant capacity.^31–36^ To the best of our knowledge, our study is the first to use reticulocytes to establish the transcript levels to investigate the impacts of HC use on the transcript levels of critical cellular redox pathway members, trying to understand how HC interacts with redox mechanisms indispensable for cellular homeostasis. It is important to highlight that this is the first study with SCA patients from Western Bahia, a region with a small migration of black people after the abolition of slavery, as well as a larger settlement of southern migrants forming its population, differentiating it from the rest of Recôncavo Baiano, which explains the peculiar miscegenation assumed by this population.^37,38^

As previously mentioned, NRF2 is a transcription factor central to redox homeostasis,^15,16,39,40^ whose mRNA levels were increased in the HC^+^ group. A similar induction pattern was reported for *AKT*, known to be responsible for maintaining the integrity of NRF2, through phosphorylation of GSK3B, a negative regulator of NRF2 by several mechanisms,^18,41^ indicating greater activation of the NRF2/ARE redox pathway in this group. Unexpectedly, we did not find higher mRNA levels of the evaluated antioxidant genes downstream of this transcription factor in the HC^+^ group, especially considering studies like Santana et al., which observed induction of the expression of antioxidant genes induced by the NRF2 pathway in PBMC and HUVEC cells.^24^

It is important to highlight that the results by Santana et al. are with PBMCs from healthy volunteers (Hb AA genotype) and HUVECs, which still have a nucleus as well as cytoplasmic organelles^42^ while here, we evaluated transcript levels in reticulocytes, which are immature forms of RBC in the final stages of differentiation (i.e., cells without the presence of the nucleus),^43^ from SCA patients. In addition to those mentioned earlier, it is also important to consider that according to Kleiveland, standard *in vivo* conditions to which cells are exposed differ from *in vitro* experiments with PBMC cell cultures.^42^

We found decreased antioxidant transcript levels of *SOD1* and *CAT* in the HC^+^ group. Previously reported studies associated greater expression and even enzyme activity (mainly for SOD) in SCD with HC use,^44–46^ like some studies in cellular models showed an increase in the enzymatic activity of SOD,^24^ with authors such as Silva et al. reporting an increase of ∼30% in CAT activity in the group with HC, *in vivo*.^35^ Therefore, we demonstrated that the high levels of *NRF2* transcripts in the HC^+^ group were not associated with a redox response in reticulocytes, as seen in Table 1, where transcription factors and their modulators did not influence the antioxidants studied. One possible explanation, as previously mentioned, may lie in the biological context, since reticulocytes, as enucleated precursors, have limited transcriptional activity compared to nucleated cells. Additionally, NRF2 might exert context-dependent functions beyond classical antioxidant gene induction, including roles in erythroid differentiation and globin gene regulation. This could be justified by other factors not analyzed in the present study that affect HbF expression since this correlation is well-documented in the literature.

*NRF2* has been reported as an inducer of HbF, as it contains a consensus sequence with the locus control region of the β-globin cluster, the DNase I hypersensitive site 2 (HS2).^47^ Thus, it forms a heterodimer with the sMAF protein and mediates looping between LCR and the ARE sequence, located 100 bp upstream of the γ-globin transcription start, activating transcription.^48^ Such action is not independent, requiring specific post-transcriptional modifications of NRF2 that do not occur without the presence of KEAP1, such as the interaction of NRF2 with the MAPK pathway,^17^ a pathway widely involved in the transmission of redox signaling.^49^

Studies have demonstrated the association of the p38 MAPK pathway with γ-globin expression in K562 cells treated with sodium butyrate and trichostatin, with p38 MAPK blocking leading to inhibition of the activation of γ-globin. Therefore, this indicates that this kinase is one of the major signaling proteins induced by drugs that induce γ-globin expression through activating transcription factors,^50^ such as NRF2.^49^ *In vitro* studies have shown that butein phosphorylates p38 MAPK, mediating this transcription factor’s activation by phosphorylation through this kinase in an adipocyte cell culture. Therefore, the dissociation of KEAP1 with subsequent translocation to the nucleus is allowed.^49^

Thus, considering the role of NRF2 in increasing HbF expression^17,47^ through signaling cascades such as p38 MAPK,^49–51^ we hypothesize that the NRF2 redox signaling pathway is triggered as part of an adaptive response, activated via p38 MAPK phosphorylation from HC use.^52^ Therefore, this leads to KEAP1 dissociation and NRF2 nuclear translocation. In addition to the change in subcellular localization, p38 MAPK can promote histone hyperacetylation, resulting in chromatin structural changes required for maximal γ-globin gene activation.^50^ However, it is important to highlight that in our data set, no significant correlation between *NRF2* levels and HbF was observed, suggesting that if such mechanisms are active, they were not detectable under the experimental conditions applied.

Another exciting result is decreased *ATF4* mRNA levels within the HC^+^ group. This transcription factor is widely documented as a critical player for translational control in response to various stress conditions and regulating physiological and pathological processes.^53^ Several studies have demonstrated that the HRI-eIF2-ATF4 pathway is necessary for erythroid differentiation by regulating the translation of globins and preventing excessive synthesis.^54,55^ Recent studies have correlated ATF4 and BCL11A, a known repressor of γ-globin genes, demonstrating that the HRI-eIF2a-ATF4 axis represses globin synthesis under heme depletion or deficiency iron conditions by promoting the transcription of *BCL11A* and consequently leads to HbF repression,^25^ to keep excess globins in line with the availability of cofactors. Therefore, ATF4 is a critical player in the mechanistic insight of fetal globin expression during cell-intrinsic erythroid stress.^56–58^

Our results, highlighted by the VIP score analysis, showed that *NRF2* was associated (increased) with the HC^+^ group, while reverse results were seen for *ATF4.* The transcript levels of the transcription factors were the variables that most depicted the studied groups, corroborating the outstanding hypothesis generated through consolidated literature, assuming patterns similar to those of the transcripts expressed at the protein level. In other words, these findings suggest a complex interplay of redox-sensitive transcription factors in response to HC, the precise biological consequences of which remain to be elucidated in future studies. Nevertheless, this study is the first to propose that HC treatment could reduce ATF4 activity, indirectly lowering *BCL11A* expression and preventing overall γ-globin repression. Additionally, HC use would trigger the NRF2-ARE redox signaling pathway via p38 MAPK phosphorylation, prompting further HbF synthesis, although this remains to be experimentally validated. Therefore, one of the main action mechanisms of HC that leads to increased levels of HbF expression could involve the NRF2 and ATF4 redox pathways as part of an adaptive response to this medication. A schematic representation of these putative regulatory mechanisms is provided in Figure 4, which should be interpreted as a working hypothesis generated from our findings and consistent with the literature, rather than as experimental evidence.

**Figure 4:**
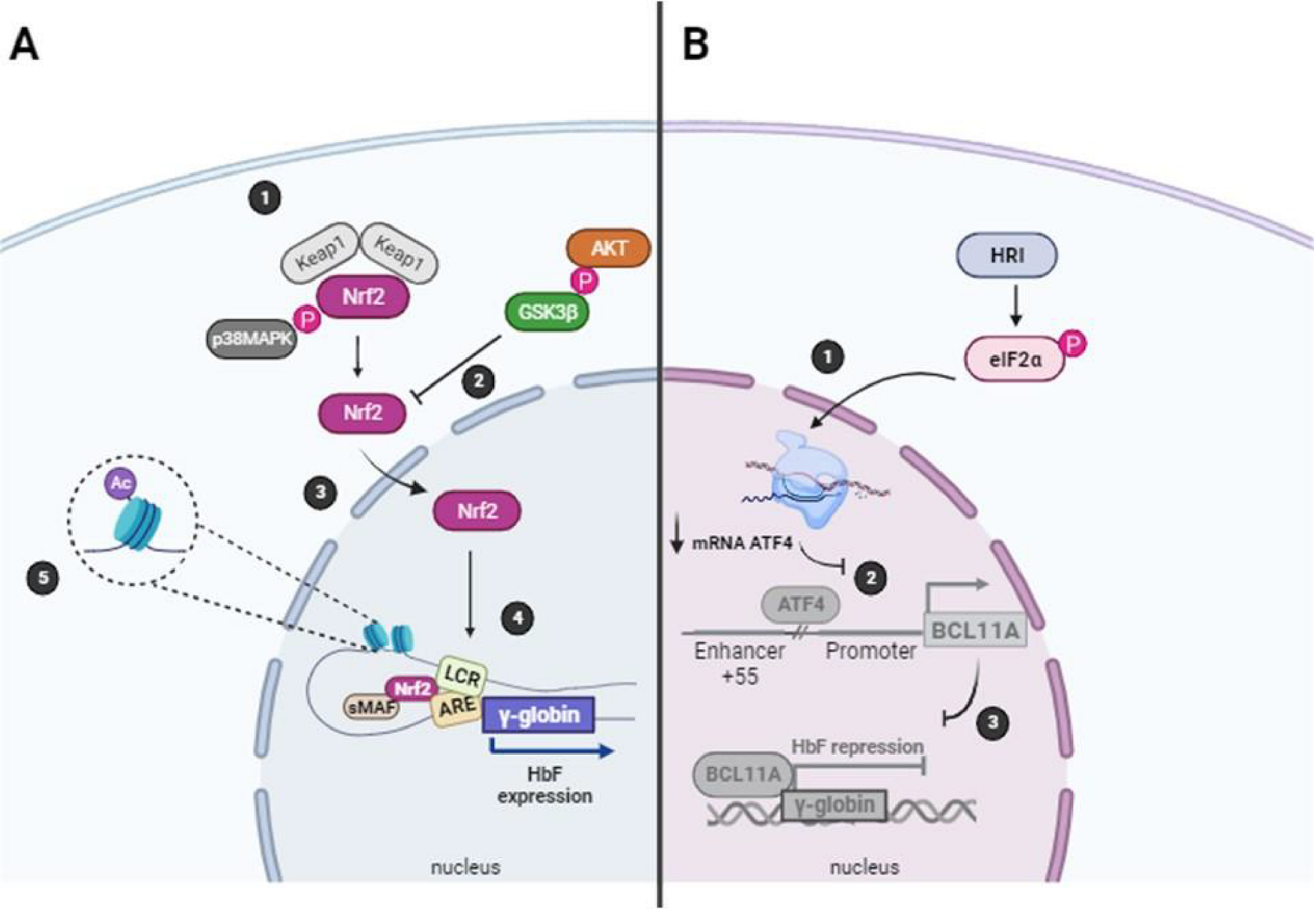
HC effects on NRF2 and ATF4. (A) 1—NRF2 activation through p38 MAPK signaling cascade and release from the Keap1-Cul3-Rbx1 complex; 2—AKT stabilizes NRF2 through phosphorylation of GSK3B, a negative regulator of this transcription factor; 3—NRF2 nuclear translocation; 4—NRF2 mediates looping between LCR and ARE, located upstream γ-globin promoters, activating Hb F transcription; 5—p38 MAPK can also promote chromatin structural changes (histone hyperacetylation) required for maximal γ-globin gene activation; (B) 1—HRI-eIF2-ATF4 pathway increases mRNA ATF4 expression; 2—ATF4 directly stimulates transcription of BCL11A by fostering enhancer-promoter contacts; 3—BCL11A represses γ-globin transcription promoting HbF repression. Created in BioRender. Venancio, L. (2025) https://BioRender.com/hgi1fss.

## 4 Conclusions

Our study, for the first time, demonstrated that the induction of the NRF2-ARE pathway stimulated by HC treatment in SCA patients was not associated with an antioxidant response in reticulocytes. This finding, along with our *ATF4* results and existing literature, suggests a potentially distinct role for NRF2 and ATF4 signaling in reticulocytes under HC treatment. This role may not involve direct enhancement of traditional antioxidant defenses. Although the involvement of these transcription factors in HbF regulation has been reported in other models, our data did not show a statistically significant association. Therefore, we propose that HC may modulate redox-sensitive signaling in reticulocytes through alternative mechanisms, which deserve further investigation, particularly concerning the noncanonical roles of NRF2 and ATF4 in erythropoiesis and HbF expression.

## 5 Material and Methods

### 5.1 Biological Samples

Blood samples (4 mL) were collected through venipuncture in EDTA tubes from SCA patients aged between 18 and 40 years, enrolled in the Sickle Cell Disease Program in Barreiras, Bahia, Brazil, located at the Leondia Ayres de Almeida health center. According to Brazilian Regulations, the Data Safety Monitoring Board (DSMB) approved this study (CAAE no. 29075620.6.0000.5026). After giving their informed consent, all patients answered a questionnaire to screen them according to the following exclusion criteria: transfusion in the last 120 days, pregnancy, bone marrow transplant, and pain on the collection day. Individuals were grouped according to whether they used HC (*n* = 10) or not (*n* = 9). We assessed, through a questionnaire, the use of other medications, the history of pain crises in the collection year, and the number of transfusions performed throughout life. After consulting the corresponding medical records, we obtained patients’ information on biochemical parameters (Table 2).

**Table 2:** Descriptive Data of Study Participants^a^

characteristics	HC^+^ (*N* = 10)	HC^–^ (*N* = 9)	*P*- value
age (years)	26.8 ± 7.17	21 ± 3.24	*p* = 0.08
*gender*			*p* = 0.04
female	6	5	
male	4	4	
*transfusion history*			Χ^2^= 3.21; df = 4 *p* = 0.52
0–20	5	7	
21–30	4	1	
31–40	1	1	
*clinical history*			
*number of pain crises in the last year*			*p* = 1
0–2	9	6	
3–5	1	3	
*number of infections in the last year*			-
0–2	10	9	
*number of hospitalizations in the last year*			-
0–2	10	9	
*mean dose*mg/kg*per day*	20 mg/kg	-	
total leukocytes	7550 ± 5.08	12750 ± 3.48	*p* = 0.21
indirect bilirubin	1.97 ± 2.23	3.30 ± 2.07	*p* = 0.34
direct bilirubin	0.65 ± 0.20	0.90 ± 1.50	*p* = 0.27
Hb F%	13.50 ± 7.69	10.55 ± 8.67	*p* = 0.76
LDH	728 ± 3.48	916 ± 5.05	*p* = 0.67

a For age, the mean value with standard deviation is indicated. The mean dose of hydroxyurea (HC) was calculated as the average dose of the group. HC^+^ refers to the group using the medication, and HC^–^ refers to the group without it. Statistical analyses were performed using one-way ANOVA for age and Fisher’s exact test for gender and the number of pain crises in the last year. Pearson’s chi-square analysis was used for transfusion history. *P* values < 0.05 were considered significant. Df = degree of freedom. Biochemical parameters were collected from patient records. The indicated values refer to the mean and standard deviation, analyzed individually by group, using hydroxycarbamide (HC^+^) and without the medication (HC^–^). Hb F%: percentage of fetal hemoglobin. LDH: lactate dehydrogenase. Statistical analyses were performed using a GLM with an ANCOVA design (corrected for age, sex, and HC dose), with *P* values < 0.05 considered significant.

### 5.2 Hemoglobin Phenotypes and Genotypes

Hb fraction quantification was obtained using high-performance liquid chromatography (HPLC) with Ultra 2 Resolution equipment (Trinity Biotech), according to the manufacturer’s instructions. We confirmed the Hb genotype in all patient samples by molecular analysis using PCR-RFLP. The PCR-RFLP reactions were standardized in a SimpliAmp gradient thermocycler (Applied Biosystems). From this, the defined reaction conditions were as follows: 10× buffer; 1.25 mM dNTP; 25 mM MgCl_2_; 10 μM of HBS1 primer (5′ GGC AGA GCC ATC TAT TGC TTA 3′), 10 μM of HBS2 primer (5′ ACC TTA GGG TTG CCC ATA AC 3′); 5 U of Platinum Taq (Invitrogen) and ∼100 ng of total genomic DNA, in a final reaction volume of 24 μL. The amplicon obtained was digested by *FastDdeI* (Applied Biosystems-Thermo Fisher Scientific, Waltham, Massachusetts, USA) restriction endonuclease, according to the manufacturer’s instructions.

### 5.3 Sample Preparation

The samples were sent to the Laboratory of Infectious Agents and Vectors (LAIVE) at the Federal University of West Bahia (UFOB), Campus Reitor Edgard Santos, for reticulocyte separation following the protocol used at The New York Blood Center. The obtained pellet was immersed in 1 mL of RNA Later solution. The RNA was extracted using the TRI reagent protocol (Sigma-Aldrich), following the manufacturer’s instructions, and its concentration was determined by spectrophotometry using a Varioskan multimode microplate reader (Thermo Scientific).

### 5.4 Real-Time PCR

The cDNA synthesis was carried out through a reverse transcription reaction using the High-Capacity cDNA Reverse Transcription Kit (Applied Biosystems-Thermo Fisher Scientific, Waltham, Massachusetts, USA), according to the manufacturer’s instructions. The primers for the genes that encode the transcription factors *NRF2* and *ATF4*, their modulators (*KEAP1*, *AKT*, and *PI3K*) and the antioxidants (*SOD1*, *CAT*, *PRDX1*, and *GPX1*), were obtained from the literature (Table S1 Supporting Information). The samples were amplified in triplicate with real-time amplification detection performed using the QuantStudio 5 equipment (Applied Biosystems) and the PowerUp SYBR Green PCR Master Mix reagent (Applied Biosystems). Except for *SOD1*, which had a temperature of 62 °C for 1 min, all other analyzed genes followed a standard melting temperature of 60 °C for 1 min. The fold change in mRNA levels was calculated using 2^–ΔΔCT^,^27^ and all the values were normalized to the expression of the *beta-actin* (*BAC*) gene.

### 5.5 Statistical Analysis

We analyzed the data using Statistica 8.0 software (StatSoft Inc., Tulsa, OK, USA). Graphics used GraphPad Prisma version 5.01 for Windows (GraphPad Software, La Jolla, CA, USA). We assessed data normality using Normal Probability Plots of Residuals for univariate analysis. Thus, all data were logarithmically transformed (log_10_) to normalize the distribution. We adopted general linear model (GLM) analysis with ANCOVA or one-way ANOVA designs. The first was adopted to compare groups because these approaches generalize to unbalanced designs with more factors, including crossed and nested and combinations of categorical and continuous variables. Thus, we established the groups (HC^–^ and HC^+^) as predictors with adjustments for gender, age, and HC dose (continuous variables). The transcript levels and biochemical parameters were established as dependent variables. The second analysis was performed to compare the age variation among the individuals. Data were expressed as the mean ± standard error of the mean (SEM) of the biological values logarithmically transformed.

We analyzed the qualitative variables, including transfusion history and the number of pain crises in the last year, using models for independent variables, specifically Fisher’s exact one-tailed test and Pearson’s chi-square test. We also performed a multivariate analysis on all markers studied (mRNA levels and clinical follow-up) based on a supervised clustering/classification method, partial least squares discriminant analysis (PLS-DA), using the MetaboAnalyst 3.0 software (http://www.metaboanalyst.ca),^28^ essentially as previously described. The PLS-DA is a multivariate classification method designed to find mathematical models that can recognize the membership of each sample in its corresponding class based on a set of measurements.^29^ As a multivariate alternative for analyzing the degree of association, the general regression model (GRM) analysis with a multiple regression design was adopted, providing more insight into the relationship between several independent and dependent variables. This analysis also provided the partial correlation, which is the individual contribution of a particular independent variable from the set to the dependent one, after controlling for all the other variables in the equation. The results of all analyses were expressed as mean ± 95% CI, and *P* < 0.05 was considered statistically significant.

## Supplementary Material



## Notes

Forward and reverse sequences of the primers employed in this study, including beta-actin as the endogenous control, the transcription factors ATF4 and NRF2, the expression modulators of NRF2 (KEAP1 and PKB/AKT), and the antioxidant genes SOD1, CAT, PRDX1, and GPX1 (Table S1); complete results obtained through a Generalized Linear Model (GLM) with an ANCOVA design, adjusted for age, sex, and HC dosage (Table S2) (PDF)
