# Supplementary material for: Hydroxycarbamide and Sickle Cell Anemia: Paradoxical Effects Related to Redox Mechanisms of Cellular Adaptation
Source: ACS Omega. 2025 Dec 9;10(50):61701–9. doi: 10.1021/acsomega.5c07897 (PMC12750237; doi:10.1021/acsomega.5c07897)
Supplement: Supplementary file 1 [file ao5c07897_si_001.pdf]

Hydroxycarbamide and sickle cell anemia: paradoxical effects related to redox mechanisms of cellular adaptation

Ilana Luize Rocha Santana[a]; Victoria Simões Bernardo[a]; Edis Belini[b]; Pâmela Lourdes Pereira da Silva[c]; Danilo Grünig Humberto da Silva[a,b]; *Larissa Paola Rodrigues Venancio[c].

[a] *Universidade Estadual Paulista Julio de Mesquita Filho, Department of Biology, São José do Rio Preto, São Paulo, BR.*

[b] *Universidade Federal de Mato Grosso do Sul, CPTL/UFMS, Três Lagoas, Mato Grosso do Sul, BR.*

[c] *Universidade Federal do Oeste da Bahia, Center for Biological and Health Science, Barreiras, Bahia, BR.*

SUPPLEMENTARY MATERIAL

TableS1. Sequences for the primers used.

Genes	*Forward primer*	*Reverse primer*
BAC	5’-CAAGCAGGAGTATGACGAGTC-3’	5’- GCCATGCCAATCTCATCTTG-3’
PRDX1	5'-TGTAAATGACCTCCCTGTTGG-3'	5'-TATCACTGCCAGGTTTCCAG-3'
CAT	5'-TGAATGAGGAACAGAGGAAACG-3'	5'-GTACTTGTCCAGAAGAGCCTG-3'
SOD1	5'-GGGCAAAGGTGGAAATGAAG-3'	5'-CAGCTAGCAGGATAACAGATGAG-3'
GPX1	5'-TTCCAGACCATTGACATCGAG-3'	5'-CACCCTCATAGATGAAAACCCC-3'
ATF4	5′-CACTAGGTACCGCCAGAAGA-3′	5′-AATCCGCCCTCTCTTTTAGA-3
NRF2	5’- GCTACGTGATGAAGATGGAAAAC - 3’	5’- AGCTCAGAAAAGGTCAAATCCTC- 3’
KEAP1	5’- AACAGAGACGTGGACTTTCG-3’	5’- GTGTCTGTATCTGGGTCGTAAC-3’
AKT	5'-TCTATGGCGCTGAGATTGTG-3'	5'-TCTATGGCGCTGAGATTGTG-3'
PI3K	5'-GGTTGTCTGTCAATCGGTGACTGT- 3'	5'-GAACTGCAGTGCACCTTTCAAGC-3'

Information genes analyzed in the study. Sequences for the forward and reverse primers of the genes used. Control endogenous beta-actin (BAC), transcription factors: Activating Transcription Factor 4 (ATF4), NF-E2 p45-related factor 2 (NRF2). Expression modulators of NRF2, Kelch-like ECH-associated protein 1 (KEAP1), protein kinase B (PKB/AKT) and phosphoinositide-3-kinase PI3K, antioxidants superoxide dismutase 1 (SOD1), catalase (CAT), peroxyredoxin-1 (PRDX1) e Glutathione Peroxidase 1 (GPX1).

TableS2. Full results GLM with ANCOVA design.

	SS	Degre of Freedom	MS	F	P
			ATF4		
Dose HC	0.027548	1	0.027548	1.464961	0.249444
Sex	0.049108	1	0.049108	2.611503	0.132060
Age	0.014975	1	0.014975	0.796353	0.389730
HC Use	0.132520	1	0.132520	7.047345	0.020997
			NRF2		
HC dose	0.13721	1	0.137208	0.148141	0.707053
Sex	0.02088	1	0.020883	0.022547	0.883135
Age	0.10249	1	0.102487	0.110653	0.7445141
HC use	6.38180	1	6.381800	6.890287	0.022182
			AKT		
HC dose	0.063069	1	0.063069	0.069392	0.797572
Sex	0.000847	1	0.000847	0.000932	0.976243
Age	0.181559	1	0.181559	0.199763	0.664436
HC use	5.078645	1	5.078645	5.587853	0.039691
			KEAP1		
HC dose	0.879590	1	0.879590	1.149853	0.306542
Sex	0.107551	1	0.107551	0.140597	0.714818
Age	0.000931	1	0.000931	0.001217	0.972797
HC use	0.118131	1	0.118131	0.154429	0.701847
			PI3K		
HC dose	0.288840	1	0.288840	2.372762	0.147450
Sex	0.001189	1	0.001189	0.009768	0.922778
Age	0.044340	1	0.044340	0.364241	0.556545
HC use	0.520956	1	0.520956	4.279552	0.059068
			CAT		
HC dose	0.095541	1	0.095541	1.369350	0.262930
Sex	0.000013	1	0.000013	0.000185	0.989356
Age	0.024257	1	0.024257	0.347661	0.565547
HC use	0.533506	1	0.533506	7.646515	0.016063
			SOD1		
HC dose	0.096107	1	0.096107	9.18669	0.008983
Sex	0.043224	1	0.043224	4.13169	0.061506
Age	0.108440	1	0.108440	10.36558	0.006176
HC use	0.109534	1	0.109534	10.47013	0.005980
			GPX1		
HC dose	0.005150	1	0.005150	0.74394	0.402934

Sex	0.063836	1	0.063836	9.22147	0.008881
Age	0.016554	1	0.016564	2.39135	0.144310
HC use	0.010831	1	0.010831	1.56467	0.231485
Error	0.096916	14	0.006923		
			PRDX1		
HC dose	0.004322	1	0.004322	0.21878	0.647167
Sex	0.015851	1	0.015851	0.80246	0.385504
Age	0.036050	1	0.036050	1.82499	0.198151
HC use	0.038433	1	0.038433	1.94561	0.184798

Full results obtained using GLM with ANCOVA design analysis (correcting for age, sex, and HC doses) of the transcriptions levels. P <0.05 was considered to be statistically significant. Value in red being statistically significant. Transcription factors: Activating Transcription Factor 4 (ATF4), NF-E2 p45-related factor 2 (NRF2). Expression modulators of NRF2, Kelch-like ECH-associated protein 1 (KEAP1), protein kinase B (PKB/AKT) and phosphoinositide-3-kinase PI3K, antioxidants superoxide dismutase 1 (SOD1), catalase (CAT), peroxyredoxin-1 (PRDX1) e Glutathione Peroxidase 1 (GPX1).
